# Nursing Student Perceptions and Attitudes Toward Patients With Cancer After Education and Mentoring: Integrative Review

**DOI:** 10.2196/27854

**Published:** 2021-09-24

**Authors:** Margot Lisa Hedenstrom, Sweta Sneha, Anusha Nalla, Barbara Wilson

**Affiliations:** 1 Wellstar School of Nursing Wellstar College of Health Sciences Kennesaw State University Kennesaw, GA United States; 2 Information Systems Coles College of Business Kennesaw State University Kennesaw, GA United States; 3 Wellstar Cancer Network Wellstar Health System Marietta, GA United States

**Keywords:** nursing students, nurse, cancer, attitudes, health care professionals, nursing, cancer patients, oncology, patient support, continuing education, mentoring

## Abstract

**Background:**

Knowledge about nursing student attitudes toward patients with cancer after an educational intervention and mentoring support is limited. This review examined the literature on this topic.

**Objective:**

This integrative review aims to explore the literature on the experiences of students who participate in an oncology elective or educational course on cancer and their attitudes toward cancer.

**Methods:**

A comprehensive search was conducted using PubMed, CINAHL, and MEDLINE databases. Each study was systematically assessed. An evidence table was completed to identify the key aspects of each study that was reviewed.

**Results:**

There is insufficient information on the impact of nursing student education on the attitudes and skills of nursing students caring for patients with cancer. An integrative review was completed on the impact of education and mentoring for nursing students on cancer care, which yielded 10 studies that were reviewed. These studies indicate that educational intervention and mentoring improve the confidence and ability of nursing students to care for patients with cancer.

**Conclusions:**

Student nurses need to be armed with knowledge, skills, and positive attitudes while caring for patients with cancer. Nursing students perform best when they have accurate information, positive role models, and mentoring by experienced oncology professionals, to support proficiency in caring for patients with cancer. The lack of knowledge of nursing students in the areas of cancer care, treatment, and patient support requires additional education and research to promote expertise and positive attitudes toward cancer and treating patients with cancer. This will support nursing students’ ability to care for patients with cancer as well as develop future educational interventions to shape nursing student attitude and knowledge. This integrative review also identifies the positive impact on the attitudes of other health care professionals who have received training or education on cancer.

## Introduction

### Background

Cancer is a significant health problem worldwide and continues to be one of the most feared diseases globally. Each year, nearly 2 million people are diagnosed with cancer and there are over 600,000 deaths in the United States [1]. The latest estimates of new cancer cases in the United States in 2020 are 1.8 million new cancer cases diagnosed as well as over 600,000 deaths in 2020 [1]. The death rate of cancer has continued to decrease by more than 2.9 fewer cancer deaths from 1991 to 2017, which results in more long-term medical and support needs for patients with cancer [1]. Cancer affects a patient’s daily life and can affect the ability to cope with the effects of illness and treatment side effects [2]. Studies have been conducted to understand the perspectives of health care professionals regarding the needs of patients with cancer or their attitudes that can be used to improve patient care and quality of life [3-9]. Nurses play a vital role in providing quality care to patients with cancer.

Nursing students need accurate information, positive role models, and mentoring to participate in caring for patients with cancer. The more nursing students are prepared, the more confidence is gained by nurses and nursing students while caring for patients with cancer. Lack of preparation by nurses can lead to anxiety, stress, and fear of caring for patients [10]. Few previous studies have examined nurses’ knowledge and attitudes regarding cancer and cancer care services. Interventions and education to help prepare nursing students for caring for patients with cancer have the potential to improve the quality of care for these patients. The results of currently available studies will provide future guidance and development of content for nursing education and practice, which will help to improve the quality of nursing care for patients with cancer and their families.

### Objectives

Formal nursing academic education has been successful in the development of a capable, trained, proficient, competent, and skilled workforce that supports patients with cancer [11,12]. It is essential that undergraduate nursing students need preparation for practice, which motivates students to care for patients with cancer competently. [7]. Nursing students have limited direct exposure to oncology and survivorship in many educational settings [13]. e-learning in the context of nursing care and oncology is an alternative method in the context of continuing education to deliver advanced nursing instruction [14]. There is limited research on the impact and influence of additional education on nursing students’ attitudes and perceptions toward oncology and survivorship. This integrative review identifies the positive impact on the attitudes of other health care professionals who have received training or education on cancer. This integrative review explores the literature on the experiences of nursing students who participate in an oncology elective or an educational course on cancer and their attitudes toward cancer.

## Methods

### Literature Search

An electronic search of literature was conducted using the PubMed, CINAHL, PubMed, and MEDLINE databases. Additional literature was obtained by reviewing the reference lists of all the identified articles. Search terms included, *cancer*, *knowledge, attitudes*, and *nursing students*. The sample size of the studies reviewed included 30-688 nurses.

In addition to searching multiple databases, citation searches were completed. The articles were manually screened by the team. Peer review by the team members was completed on the articles that were found and summarized, as well as a summary of the articles identified. Reviews were excluded. Owing to the lack of articles on all types of health care personnel in the literature, although nursing students were the focus of the review, additional types of health care students (physicians) were included in the final articles for the study, which further identified a need for additional research in this area related to nursing students.

This integrative review will explore nursing students’ perceptions about caring for patients with cancer and survivorship and help identify the needs for further education and intervention.

### Data Evaluation and Analysis

The selection process resulted in 10 articles with a wide range of methodological approaches. Multiple articles were reviewed, and relevant articles were synthesized. The authors independently appraised the studies and discussed them. The studies were rated on a scale of high or low [15] related to the relevance of the study to the purpose of the review. An evaluation was performed using the Johns Hopkins tool for review [16]. Studies were also reviewed for research design, methods, and data analysis. The final information yielded 10 relevant studies. A dissertation that was a quantitative study by Burns [17] was reviewed as this initial research has been used as a tool to measure knowledge and attitudes toward cancer. Two tools by Burns [17] and Haley [18] were used in subsequent studies [3,17-22].

The studies were reviewed and scored to evaluate the inclusion criteria. The Johns Hopkins Evidence-Based Practice tool was used. The inclusion of the team helped strengthen the results via multiple content expert analysis.

## Results

### Literature Search

Articles that were screened and evaluated for inclusion comprised studies that were qualitative and quantitative research articles published in the period between 1981 and 2019. Commentaries and books were excluded. The titles and abstracts were screened to fit the inclusion and exclusion criteria. The final screening narrowed down the total information to 10 articles after exclusion of nonrelevant articles.

This integrative review identified 10 studies focused on nursing students’ attitudes toward knowledge about cancer, attitudes, and experiences of caring for patients with cancer. Studies involving nursing students and multidisciplinary professional groups containing students were included.

**Figure 1:**
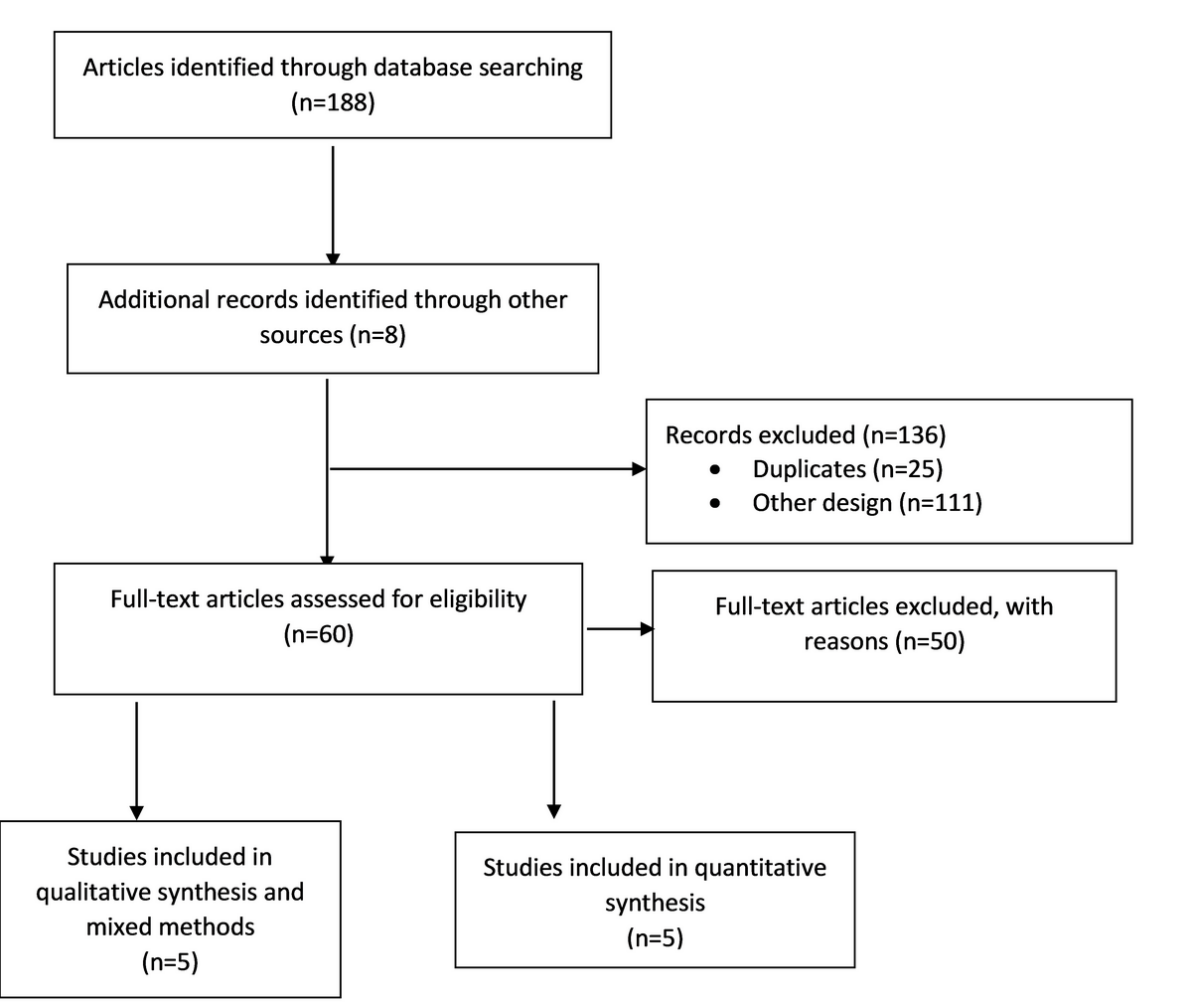
PRISMA (Preferred Reporting Items for Systematic Review and Meta-Analyses) flow diagram.

The search was carried out in October 2019 and included all the results from the databases up to that date. All articles written in English were also included. A PRISMA (Preferred Reporting Items for Systematic Review and Meta-Analyses) [15,23] flow chart was used to search the relevant literature (Figure 1).

The lack of consistency in the measurement of cancer attitudes was a finding in this review. Various studies have attempted to address face and content validity, although the approaches and questions varied between most of the studies. There is limited research on the knowledge and attitudes of nursing students in the literature. In addition, the lack of research in this area supports the gap in measuring and addressing the knowledge and attitudes of health care providers in caring for patients with cancer.

### Data Evaluation and Analysis

The selection process resulted in 10 articles (Table 1) with a wide range of methodological approaches. Again, team members reviewed the articles based on the Johns Hopkins tool and ranked articles using a high or low scale for consideration for inclusion and the processes outlined in Whittemore and Knafl [24].

As the studies were reviewed, key areas were noted in the review process that will be fully described in the following sections. These areas included nursing students’ attitudes and knowledge of caring for patients with cancer, factors that affect nursing students toward caring for patients with cancer, nurses and health care professionals’ attitudes toward cancer, work experience in cancer care, and education and training on cancer care as key areas identified.

**Table 1:** Articles and methodological approaches.

Study (country)	Key points	Design and sample	Notes and discussion
Dedeli et al, 2016 (Turkey) [20]	Generally, there was a positive attitude toward cancer. Nurses who had more experience with cancer care and who had more experience in caring for patients with cancer showed attitudes that are more positive.	Descriptive; sample of 332 nurses; used Turkish version of the attitudes toward cancer scale	Recommendation was that experienced nurses should care for patients with cancer.
Cunningham & Bater 2017 (United Kingdom) [4,5]	In this study, 2 sets of participants’ attitudes in 2003-2004 and 2016-2017 are compared. Current nursing students exhibited positive attitudes in cancer care.	Convenience sample of 152 students in 2003 and 154 students in 2016; questionnaire provided and optional interview taken	In second study, mentoring and clinical experience are more common and more positive attitudes were noted in second cohort.
Edwards et al, 2016a (United Kingdom) [6]	Two key findings in this study are (1) students were interested in meeting patients with cancer outside clinical environment and (2) lecture and practical experience with clinicians and patients are important for developing confidence and competence of students in cancer care.	Descriptive, explorative, and qualitative design; semistructured interviews with 12 student nurses and 7 stakeholders. The interviews were audiotaped, transcribed, and analyzed using content analysis.	This study helped support the need for further development of cancer education experiences for nursing students based on the responses and themes identified through the research study.
Edwards et al, 2016b (United Kingdom) [7]	Study evaluates undergraduate curriculum content on cancer education.	Intervention study had two groups: 84 participants in intervention group and 91 in comparison group. Pre-post test was administered.	Study found that new model to deliver cancer education may improve knowledge, attitude, and confidence regarding cancer by student nurse population in study. Study recommends further development and research.
Felton et al, 1981 (United States of America) [8]	Purpose of the study was to measure attitudes toward cancer pre and posteducation intervention.	Pre- and posteducation sample of 545 nurses and nursing students on cancer attitude survey	Study supports need for educators to identify attitudes and plan education to help support positive attitudes.
Hsu et al, 2019 (Taiwan) [25]	Study supports implementing education intervention to increase nursing student knowledge, confidence, and skill in cancer care.	Random study with 213 students using 2 group pretest and posttest surveys.	One group received outcome-based program with simulated exercises and the control group had objective-based education, which was traditional design. Experimental group had educator with more experience and outcome-based simulation included.
Kapucu et al, 2018 (Turkey) [10]	Nursing students felt working with patients with cancer was *difficult*.	Focus groups with 61 participants and survey questionnaire with 129 participants; mixed methods study	Authors recommend additional research and focus on cancer care and ways to support patients with cancer.
Kav et al, 2013 (Turkey) [2]	Nursing students felt care was difficult and expressed fear and uncertainty in caring for patients with cancer.	Described study with questionnaire n=167 and 2 focus groups of nursing students as participants	Student participants suggested need for additional orientation and clinical placement to prepare for cancer patient care. Mentors were helpful including preceptors and nurses in learning to care for patients with cancer.
Powell et al, 2019 (Canada) [11]	The students had an observation experience in their first year nursing before clinical experience to help learn about cancer and perspectives of cancer care.	Qualitative: 10 first-year nursing students participated in individual semistructured interviews	This study felt that low-risk observation experience was an opportunity for students to learn about cancer care and nursing roles in treating cancer.
Sharour et al, 2017 (Jordan) [12]	Age was noted as an influencing factor in the participants’ attitude, as younger study participants had more negative attitudes toward cancer and death and dying patients with cancer. Less experienced students had more negative attitude and responses.	Descriptive study with 100 nursing students participating; one time survey of students on Frommelt Attitude toward care of the dying scale and death attitude profile-revised scale	Study conclusions note that training and education can help support knowledge and attitudes. Nurses in the study with more experience and training had more positive attitudes noted.

### Nursing Students’ Attitudes and Experience With Cancer and Caring for Patients With Cancer

Nursing students and nurses’ attitudes have been infrequently studied. Four studies specifically attempted to evaluate nursing student attitudes through a variety of interventions. Kapucu and Bulut [10] found that 80.6% of student nurse participants felt it was difficult to work with patients who have cancer, whereas 85.3% stated it was difficult to provide care to patients with cancer. Two studies [7,8] measured students’ attitudes after providing an educational intervention. In another study, academic levels and age of the students were the determinants of the study [20]. The more experience an individual had, the higher was the positive perception toward cancer [20]. In two studies [2,10], most students expressed fear, hopelessness, and uncertainty.

Kav emphasized that students need to overcome fear, hopelessness, uncertainty, and the association of cancer with death. Implementing orientation programs, meeting the professional oncology team and communication on past and present experiences, increasing the time of clinical practice placements, and motivational activities are needed for students to gain more confidence and remove fears in caring for patients with cancer [2]. All studies recommend further research in the area of nursing student support and education to improve nursing students’ confidence and ability to care for patients with cancer.

### Factors That Affect Nursing Students Toward Caring for Patients With Cancer

Nursing students reported that their feelings toward patients were negatively affected because of poor communication, lack of confidence, and difficulty in providing physical care [10,25]. Students’ lack of knowledge and experience, fear of patients and family members, and fear of pain were reasons for the students’ negative feelings [10]. Therefore, it is recommended that students receive better training on patients with cancer and the needs and issues of their care [10]. The skill of supporting patients during cancer care was recognized as a skill that can be developed through education and exposure to patients with cancer and families through direct experience.

One study identified that students felt formal education as well as partnering with cancer clinical experts and patients with cancer and their families was important to help them feel confident and prepared to help care for patients with cancer [6]. A significant relationship was identified between nurses’ attitudes toward cancer and factors such as age, gender, years of experience in oncology, and support working with experienced staff. The results of a study of over 300 nurses suggested that female nurses aged >40 or with >10 years of experience displayed positive attitudes toward patients with cancer [20].

Another study conducted in 2006 [4] found that students who were initially worried about cancer identified that past experiences and having clinical support and education positively influenced their outlook of cancer and caring for patients with cancer. The same study was repeated 10 years later, with another cohort of students that supported the initial finding that the positive perceptions of caring for patients with cancer continued through education and support interventions. Confidence in providing care by students increased in a later study from 62.2% in 2004 to 75.34% in 2017. In addition, perceived skill in providing care by students also increased, as in 2003, 77.6% felt they lacked skills to care for patients with cancer, whereas in 2017 only 37% felt they lacked skills to care for patients with cancer. Changes in beliefs and attitudes were noted, and students reported increased confidence with a positive outlook and attitude toward caring for patients with cancer. The second cohort of nursing students was found to have a different work environment than a decade ago. Previously, mentor support and additional learning in clinical areas were not as prevalent. The first cohort helped mentor the second cohort by providing advice and support [5].

On the basis of these findings, an increase in awareness and knowledge of the cancer disease and treatment continuum may support increased effectiveness in preparing students to treat patients with cancer [5]. The student nurses reported that they were fearful, worried, and experienced feelings of pity when providing care for patients experiencing pain or who had a terminal illness. A common perception among students is that cancer is often a terminal illness that prevents students from working in oncology settings [10,25]. These studies indicate that efforts are needed to educate nursing students and nurses in the area of cancer care.

### Nurses and Health Care Professional Attitudes Toward Cancer

The studies presented in this review noted that nursing students and health care professionals often have negative attitudes toward cancer and caring for patients with cancer. Clinical decision-making is a challenge for health care professionals when they express negative attitudes toward cancer [21,25]. This study proposed that educational programs could remove fear and create a positive image of cancer in health care professionals. In addition, this study identified the need for education to help support and improve attitudes toward cancer to provide high-quality care to patients.

Understanding and guiding health care professionals’ behaviors in a positive direction can improve the quality of care of patients and families. The experience of both physicians and nurses correlated positively with improved attitudes toward caring for patients with cancer. In contrast, less experienced professionals were more afraid of cancer and death. Efforts are needed to educate health care professionals on the psychosocial aspects of terminal care for patients with cancer.

### Work Experience in Cancer Care Setting

Nurses’ attitudes toward patients with cancer are affected by many factors such as age, years of experience in oncology, gender, knowledge of the disease process, and clinical experience in cancer units. In addition, more experienced nurses had a positive attitude toward patients with cancer and helped patients cope with chemotherapy, radiation therapy, or any complications associated with cancer treatment [20]. According to Sharour et al [12], younger students expressed negative attitudes toward patients with cancer. Increased support through academic and formal training has been shown through these studies to positively impact attitudes toward patients with cancer. Confident and skilled nurses are able to manage patients in difficult situations. Improving support, knowledge, and education for nursing students are critical elements that will improve communication and eliminate fear while caring for patients with cancer.

### Education and Training on Cancer Care

Lack of knowledge of cancer care among students is evident in the literature. Effective clinical classroom and clinical education aspects are crucial for improving nursing students’ attitudes toward caring for patients with cancer. Such education should include information on cancer treatment and survivorship and the assigned time spent in various oncology settings. Key components for success include a strong orientation to cancer care and clinical cancer care settings, mentorship and role modeling by staff and teachers, feedback and communication during cancer care experiences, elective oncology courses in nursing programs, and faculty support for students to support the development of competency in caring for patients with cancer [10,25]. Oncology content should be required, and elective courses should be offered to help further develop skills and expertise in cancer care and treatment. Students should be partnered with strong clinical expert mentors who are clinical experts who also provide a positive supportive environment to learn the nuances of cancer care. In addition, an observational educational experience was identified to improve nursing students’ knowledge [11,25]. Through this observation and study, students gained a new perspective on cancer care.

Nurses and student nurses who received training reported positive perceptions when caring for patients with cancer. Students feel that meeting patients with cancer outside the hospital setting might help them to create more confidence in talking and managing patients with cancer. The inclusion of cancer content in the undergraduate curriculum supports nursing students’ ability to develop positive attitudes while strengthening their knowledge base while increasing experience and confidence in caring for patients with cancer, which will improve quality of care.

### Summary

There is a lack of literature highlighting cancer education and clinical experience as an intervention in the undergraduate nursing student population. There is also a lack of literature that evaluates the impact of such education on the care delivery experience in cancer post intervention. The skills and knowledge gained through such an intervention could improve nursing student confidence and quality of care. Additional research would help to identify and address what specific training is needed to help improve nursing students’ skill levels and attitudes toward cancer care [6,7]. Important findings from the studies include the recommendation that nursing students benefit from participation in the classroom and clinical experiences to improve knowledge and skills in oncology nursing. Oncology content should be included in the core curriculum of undergraduate programs. In addition, students should work with oncology nurse mentors who are clinical experts and bedside educators to provide positive learning experiences [13,25]. Specific directed education of nursing students as well as formal research to measure the impact of education and training on cancer care and the impact on nursing students and patients is needed.

## Discussion

### Principal Findings

As noted in the studies and key findings, many nursing students experience a variety of feelings, including fear, hopelessness, anxiety, and being unprepared to provide care to patients with cancer. The outcomes of these studies indicate that education plays a key role in alleviating fear, hopelessness, and promoting confidence in nurses and nursing students in taking care of patients with cancer. In the articles found in the study, key themes of the need for education, mentorship, and support of nursing students were key findings. The few published studies confirm the noted improvement in students’ confidence in caring for patients with cancer after formal educational support and mentoring. There is limited availability of these types of studies within the literature as evidenced by this integrative review.

### Limitations

There are limitations in the literature as well as in this review. The inclusion of health care professional students was incorporated into the study to identify the key findings of education on cancer care. The needs of nursing students warrant further exploration to determine what needs nursing students have compared with other disciplines. In addition, the limited availability of articles increases the challenge of fully outlining the impact of education on nursing student attitudes. Additional manual search of articles could have potentially strengthened the study if additional time could be provided to continue citation searching and potentially expand search terms for the study. Although there is a lack of research studies, the impact of education and mentoring support was a finding available in these few studies.

Most studies have focused on performing research in one setting. A broader focus in scope and multiple institutions are required, which would include many types of institutions and multiple countries. Several investigators have acknowledged that single-survey instruments alone are not always appropriate for analyzing the nature of attitudes of nursing students. Few tools and formal programs were found to support nursing students in their training to care for patients with cancer in the curriculum. In addition, there is limited research overall on the subject area of nursing students and their attitudes toward patients with cancer. With some undergraduate programs, there is limited information and focus on the care of oncology patients.

### Comparison With Previous Work

Working with patients who have cancer and their families can be challenging because of the complexity of patient care needs in this population. One study noted a significant relationship between nurses’ attitudes toward cancer and factors such as age, gender, years of experience in oncology, and support at work from experienced staff [20]. The results suggested that nurses aged >40 years or those with >10 years of experience displayed positive attitudes toward cancer [20].

Health care professionals have expressed negative attitudes toward cancer, and changing these attitudes has been noted to be challenging [10]. Kapucu and Bulut [10] explained that most of the students in the study expressed sorrow, worry, and pity and were not psychologically ready to provide care for patients with cancer. Moreover, 80% of the students believed it was difficult to work with patients who have cancer and care for them. Because of this study, a training course in cancer care was added to the nursing curriculum to improve knowledge in cancer care [10].

Cunningham and Bater [5] asserted that observation experiences in oncology clinics are important for students to gain experience and be ready to care for patients with cancer. Kav et al [2] reported that effective student orientation programs, organizing meeting share experiences, motivating activities, and role modeling by staff and teachers help students to communicate efficiently with patients who have cancer. In another study, Cunningham and Bater [4,5] compared the results of student nurses’ experiences of caring for patients with cancer between 2003 and 2004 and repeated in 2016-2017. There is a significant shift in between the results. The overall experience of caring for patients with cancer increased from 66% in 2003-2004 to 75.34% in 2016-2017. In addition, differences were noted in the confidence level in caring for patients with cancer, which increased from 34% to 96%. Another interesting difference noticed in this study is that the number of females enrolled in oncology nursing increased and the male workforce decreased in the span of 10 years. The current generation student nurses have more support in terms of mentoring, clinical education, and theoretical knowledge compared with the previous students. Younger and female students both showed negative attitudes and emotions compared with senior and experienced nurses. This study indicated that the level of experience and qualification is an important criterion in caring for patients with cancer. Highly qualified nurses can manage patients with cancer in difficult situations [12].

A new model to deliver cancer care education on cancer and survivorship was introduced by Edwards et al [6,7], where participants were divided into intervention and comparison groups and provided with different cancer education programs for 3.5 days and 2 days. The intervention group demonstrated more positive attitudes toward caring for patients with cancer and more confidence in their ability to deliver cancer care related to the comparison group. This new model for the delivery of cancer education focused on survivorship through involvement with patients, families, and oncology clinical experts may improve knowledge attitudes and confidence in delivering cancer care [6,7].

Almost all the reviewed studies were conducted with Turkish and UK nursing students or nurses. Therefore, the results are not generalizable because of the cultural differences and notable differences in education programs [13]. In addition, studies that evaluate educational and mentoring interventions in cancer care for nursing students and nurses who are not homogenous subjects have not been completed. Identifying strategies and educational needs for cancer care in nursing students and nursing populations remain as gaps in the literature. Future research is needed to identify and further develop specific educational and clinical opportunities that support positive attitudes and improve skill levels for cancer care in nursing students [13].

However, there are limited studies in the United States on undergraduate nursing students toward caring for patients with cancer. Many international studies were found on this topic. In summary, key themes that were identified in the review of the literature include lack of training for health care professionals, including nurses on cancer care and survivorship, and lack of measurement of nurse and health care attitudes. A few international studies [2,6,7,10,21] highlighted an educational intervention and impact on nursing attitudes. These studies indicate that educational interventions such as initial orientation programs, regular meetings, and sharing experiences, including mandatory oncology courses in the nursing student’s curriculum, collaboration with patients, and support from physicians and senior nurses help to support cancer care knowledge and attitudes by health care professionals in these studies.

### Implications for Research

This review found little research on the attitudes of nursing students or nurses toward cancer after being provided an educational intervention. Further study of the impact of educational programs and training on student nurses and nurses can help identify what improvements can be made in the education of nursing students and nurses. Owing to the large incidence of cancer, nursing attitudes and knowledge need further study. On the basis on the findings and lack of research, a descriptive study is recommended to investigate nursing students’ perceptions about cancer and cancer survivorship.

### Implications for Education and Practice

Improving the attitudes of undergraduate nursing students helps them become more confident in caring for patients with cancer. Mentoring nursing students through classroom education and access to experienced practicing oncology nurses can strengthen the skills of nursing students [13]. The variety of roles of oncology nurses in the inpatient and outpatient settings offer great possibilities to help expose nursing students to cancer care by nursing experts. Oncology nursing education can be incorporated into the curriculum through a variety of education or observation experiences [11]. In addition, faculty, experienced oncology nurses, and other health care professionals with experience in caring for patients with cancer can provide extra support to student nurses. Educational programs can increase knowledge about cancer care and treatment, while decreasing fear and improving student nurse skills and confidence. Oncology nursing courses that provide knowledge, skills, and attitudes toward caring for patients should be included in nursing curricula.

There is some research in the literature on health care professionals’ attitudes toward caring for patients with cancer; however, there is limited research on nursing care. Further exploration of additional content on cancer will help prepare future nurses caring for patients with cancer. There is also the potential for incorporating electives to allow students to further expand their knowledge of cancer care (Textbox 1).

**Best practices for allowing students to expand their knowledge.**

**Best practices**

- Increase the amount of educational training and clinical rotations for nursing students
- Mentor undergraduate nursing students by placing them with experienced oncology nurses. Offer internships and other clinical programs to increase exposure to cancer care
- Offer elective oncology classes as part of the curriculum for undergraduate nursing students

### Knowledge Translation

Experienced oncology nurses can support student nurses through mentoring and sharing knowledge and skills with student nurses in clinical and classroom settings. Inclusion of certain electives and updating the curricula of nursing programs could potentially impact nursing student attitudes toward cancer, as has been noted in other disciplines. The studies reviewed support education and clinical experiences to help increase knowledge and alleviate anxiety in caring for patients with cancer (Textbox 2).

To help prepare and support students, faculty can support students in self-assessment on their educational needs and feelings about caring for patients with cancer. Students can be supported through educational interventions, access to experienced nurse faculty and practicing experts in cancer care to help provide mentorship and support to students in their journey in caring for patients with cancer. Expanding current strategies such as increasing clinical or observation experiences and additional formal training can be used to address knowledge gaps and fear for inexperienced student nurses new to the oncology field.

**Clinical experiences to help increase knowledge translation.**

**Knowledge translation**

- Nursing attitudes toward cancer using an educational intervention is perceived to improve attitudes but has had little research
- Inclusion of electives and education into nursing programs could potentially impact nursing student attitudes toward cancer as has been noted in other disciplines
- Nursing students often receive little education on oncology and often feel unprepared in caring for patients with cancer

### Conclusions

Efforts must focus on identifying, developing, and testing interventions to improve nurses’ attitudes and their expertise in cancer care. Previous research has noted the need for additional studies to be conducted as well as the need for a nursing curriculum to include education to improve attitudes to better understand what interventions support the expertise needed to provide high-quality care to patients with cancer. Furthermore, it appears that nursing students recognize the need for additional training through the common themes of fear and lack of confidence in supporting people with cancer. A preceptor-based clinic teaching method is another innovative way to train and create a positive attitude toward patients with critical ailments. There have been efforts and exercises conducted in this area, such as the clinical teaching blended learning program, which was conducted from September 2019 to December 2019 and included 150 nurse preceptors [26]. Although there are innovative methods that aim to focus on improving nurses’ attitudes and expertise toward patients with chronic conditions, the area still needs a significant impetus. This will take time, attention, and focus to help strengthen cancer care expertise in the nursing student population.

Patients and caregivers impacted by cancer need to be able to manage the short- and long-term effects of cancer treatment and the long-term impact of cancer. Cancer cases are approaching 2 million patients per year, with increases noted in the survivorship of patients with cancer. As patients with cancer are prevalent in all parts of the health care system during and after treatment, nursing students and nurses need to have expertise, experience, and confidence to provide high-quality care and support patients and families of patients living with cancer. Undergraduate nursing education can provide a differentiating position to help prepare nurses to support patients with cancer and to improve the quality of life for those impacted by cancer. The studies included in this integrative review studied general attitudes of health care personnel toward cancer or death and dying. In studies that evaluated professional attitudes, the need for additional education and support from health care personnel was a common theme. Little research on attitudes or the impact of an educational intervention on attitudes is present in the literature. Further research is needed to determine effective strategies to increase competency in cancer care for nursing students and to understand the attitudes of nursing students. This would help identify innovative educational strategies that are effective in increasing the knowledge and attitudes of nursing students and nurses toward patients with cancer.

## Abbreviations

PRISMA: Preferred Reporting Items for Systematic Review and Meta-Analyses
